# Sentiment Measured in Hospital Discharge Notes Is Associated with Readmission and Mortality Risk: An Electronic Health Record Study

**DOI:** 10.1371/journal.pone.0136341

**Published:** 2015-08-24

**Authors:** Thomas H. McCoy, Victor M. Castro, Andrew Cagan, Ashlee M. Roberson, Isaac S. Kohane, Roy H. Perlis

**Affiliations:** 1 Center for Experimental Drugs and Diagnostics, Department of Psychiatry and Center for Human Genetic Research, Massachusetts General Hospital, 185 Cambridge St, 6th Floor, Boston, MA 02114, United States of America; 2 Partners Research Computing, Partners HealthCare System, One Constitution Center, Boston, MA 02129, United States of America; 3 Department of Medicine, Brigham and Women’s Hospital, Suite 255, New Research Building, 77 Avenue Louis Pasteur, Boston, MA 02115, United States of America; University of Oxford, UNITED KINGDOM

## Abstract

Natural language processing tools allow the characterization of sentiment–that is, terms expressing positive and negative emotion–in text. Applying such tools to electronic health records may provide insight into meaningful patient or clinician features not captured in coded data alone. We performed sentiment analysis on 2,484 hospital discharge notes for 2,010 individuals from a psychiatric inpatient unit, as well as 20,859 hospital discharges for 15,011 individuals from general medical units, in a large New England health system between January 2011 and 2014. The primary measures of sentiment captured intensity of subjective positive or negative sentiment expressed in the discharge notes. Mean scores were contrasted between sociodemographic and clinical groups in mixed effects regression models. Discharge note sentiment was then examined for association with risk for readmission in Cox regression models. Discharge notes for individuals with greater medical comorbidity were modestly but significantly lower in positive sentiment among both psychiatric and general medical cohorts (p<0.001 in each). Greater positive sentiment at discharge was associated with significantly decreased risk of hospital readmission in each cohort (~12% decrease per standard deviation above the mean). Automated characterization of discharge notes in terms of sentiment identifies differences between sociodemographic groups, as well as in clinical outcomes, and is not explained by differences in diagnosis. Clinician sentiment merits investigation to understand why and how it reflects or impacts outcomes.

## Introduction

The use of large-scale electronic health record data has enabled powerful, efficient investigation of clinically important questions ranging from pharmacovigilance to risk stratification[1]. However, coded data such as diagnostic and billing codes provide a limited window into clinical status. Electronic health records often capture provider decision making and analysis as unstructured data, such as narrative notes. These data are appealing in their richness but have been difficult to quantify, particularly with the efficiency required for health-system-wide analysis.

Even brief fragments of text may reflect the feelings of the author about a given topic. That is, some words or groups of words reflect more positive ('excellent') or negative (‘horrible’) valence. Recently, tools have been developed and validated to facilitate the algorithmic quantification of these feelings, referred to as sentiment, in text documents[2]. This approach has found diverse application in health-related topics, allowing investigation of the correlation between happiness, geographic regions, and health on Twitter[3], impact of online discussions of cancer[4], attitudes towards tobacco products[5], and proxy measures of satisfaction with health care[6] or health care reform[7].

We hypothesized that sentiment could be measured in narrative notes written by clinicians, and that these data might provide insight into patient characteristics not explicitly captured by claims codes or diagnoses, as well as clinician impressions of, or attitudes toward, individual patients. In particular, if these data, not typically captured at a clinical population level, were shown to have predictive validity, it could prompt greater effort to utilize narrative notes to augment coded data sources.

We therefore applied a sentiment scoring algorithm, also known as opinion mining, to quantify sentiment in a corpus of narrative hospital admission and discharge notes. We examined differences between patient subgroups, as well as the association between sentiment measures and clinical outcomes as a test of predictive utility for this approach.

## Materials and Methods

### Cohort and outcome derivation

We used Informatics for Integrating Biology and the Bedside, or i2b2, server software (i2b2 v1.6, Boston, MA, USA)[8] in order to access and parse data from the electronic health records of a large Boston-based hospital. The i2b2 software [9, 10] is an informatics framework deployed at more than 100 academic health centers internationally for managing human health data. Data available in the hospital EHR include sociodemographic identifiers, billing codes, laboratory results, problem lists, medications, vital signs, procedure reports, and narrative notes. The present analysis focused on sociodemographic features, billing codes, and narrative admission and discharge notes written by staff physicians. (Inclusion of year of service in models did not meaningfully change results and is not addressed further.) The specific features examined in regression models were standard sociodemographic and clinical cohort descriptors selected by the authors a priori as well as those shown in our prior work to be associated with readmission[11, 12].

In order to examine the extent to which features of clinical notes were specific to a given patient population, two different cohorts were drawn from the records of this health system. The first included all patients admitted to a 24-bed inpatient psychiatric unit between January 2011 and January 2014. The second included all patients admitted to a general medicine unit spanning multiple floors in a large teaching hospital during the same period. As adult services, only individuals age 18 or older were included; no other inclusion or exclusion criteria were applied. The Partners Institutional Review Board approved all aspects of this study.

The primary outcome measure of interest in the psychiatric cohort was time to psychiatric or all-cause hospital readmission, determined by examining the period following index discharge to identify subsequent admissions. For the larger, general medical cohort, in addition to time to readmission, time to all-cause mortality was also examined. (The small number of events in the psychiatric cohort precluded analysis of mortality in this group.)

### Development and application of natural language processing tool

Multiple methodologies have been developed to characterize aspects of text in a high-throughput fashion [13–16]. In general, two conceptually different approaches exist: one based on machine learning in which a model of word or phrase usage is fit to documents of known sentimentality and then applied to sample documents, and another in which a curated lexicon of subjectivity and sentimentality is used to score words occurring within a sample document. As we are unaware of a “gold standard” corpus of medical notes rated for sentiment, we necessarily used the second approach. In particular, we used Pattern (v2.6), an open source implementation of lexical opinion mining developed at the University of Antwerp [17–19]. As the Pattern library is freely available under the Berkley Source Distribution (BSD), the source code is the definitive description of the method used. In brief, this method depends on matching words and phrases to an included lexicon of nearly 3,000 words annotated for polarity (negativity vs positivity rated -1 to 1), subjectivity (not subjective to subjective rated 0 to 1), intensity modifier (1/2 to 2x) and negation (reverses polarity)[20]. In this approach unrecognized words–those not included in the lexicon–are ignored. The score for a given document is the mean of all recognized words after accounting for preceding negation (inverting polarity) and multiplying by the intensity modifier for relevant adjectives. This approach has demonstrated accuracy of 75% versus a gold standard corpus of movie reviews[21, 22]; to our knowledge, its performance in a corpus of clinical notes has not been previously examined, and no manually-classified corpus of clinical notes is available. Consistent with the work of Mitchell[3], no effort is made to tie these scores to particular topics. Instead, we estimate the aggregate positivity and negativity, and the subjectivity versus objectivity, for a given narrative note.

### Analysis

As primary measures for analysis, we selected the product of two estimated characteristics—that is, to what extent are terms subjective (1) versus objective (0) and positive (+1) or negative (-1)? For each narrative note, this yields 2 scores: subjective positivity, in a continuous range from 0–1, and subjective negativity, in a continuous range from 0–1. Thus, an entirely neutral note would be scored a 0; a note comprised entirely of subjective positive statements would be scored a 1. These two features (positivity and negativity) were examined separately, rather than treated as a continuum, to allow for the possibility that some notes might have high levels of both—i.e., more sentiment-expressing words of both polarities.

Associations between the product of positive or negative sentiment and subjectivity, and sociodemographic or clinical features, were examined using mixed effects models, in order to account for presence of multiple clustered observations (i.e., multiple hospitalizations) per patient. (In sensitivity analyses utilizing only the index (initial) observation, results were not meaningfully different and are not presented here.) Normality assumption for independent variables was confirmed using visual inspection of distribution plots and by Kolmogorov-Smirnov test; Charlson comorbidity index was accordingly log-transformed as in our prior investigations. Linearity of relationship between individual independent variables and sentiment was confirmed by visual inspection of residual plots, and by inclusion of quadratic terms. Homoscedasticity assumption was confirmed by visual examination of a plot of the standardized residuals against regression standardized predicted values. Models were estimated using default settings of the xtmixed package in Stata 13.1 (Statacorp, College Station, TX), including independent covariance matrix structure and model fit via maximum likelihood. Tables present fully-adjusted effects (i.e., adjusted for all other terms in the model) because the high risk of confounding limits interpretability of crude effects. Time to readmission used survival analysis with results censored at readmission, death, or end of available follow-up period, whichever came first. Effects of sentiment scores were examined using Cox proportional hazards models, adjusted for sociodemographic and clinical features, after confirming that the proportional hazards assumption was met for each independent variable by formal test and by using visual inspection of Schoenfeld residuals.

This study obtained IRB approval from the Partners Human Research Committee under protocol number 2012P002527. No informed consent was required, as this project is a retrospective health care utilization/clinical study involving thousands of patients and multiple years of data—that is, consent could not be feasibly be obtained from all subjects.

## Results

### Psychiatric Cohort

For the 2,010 individuals with 2,484 discharge notes (mean 1.24 SD 0.72 discharges per patient; 84.5% with a single discharge) from a psychiatric inpatient unit, 49.1% were male and 71.3% Caucasian by self-report (S1 Table); the remainder described themselves as black (9.5%), Asian (4.3%), Hispanic (10.6%), and other (4.3%). A total of 54.6% had public insurance, and 58.2% were admitted via the emergency department rather than directly to the unit. Primary psychiatric diagnosis was a psychotic disorder (schizophrenia, schizoaffective disorder, or psychosis not otherwise specified) in 18.9%; the remainder were mood or personality disorders. Mean age in this cohort was 43.8 years (SD 16.4); mean age-adjusted Charlson comorbidity index was 3.3 (SD 4.5).

Table 1 summarizes the mixed effects model examining the association between positive (left columns) and negative (right columns) sentiment and individual sociodemographic and clinical aspects of the cohort. Sentiment was generally similar between discharge notes for genders and across racial groups, with the exception of notes for individuals who self-identified as Hispanic, in which significantly greater levels of both positive and negative sentiment were detected. Public insurance was associated with significantly lower levels of positive sentiment; greater medical comorbidity was associated with lower levels of positive and negative sentiment; and presence of a psychotic disorder was associated with greater negative sentiment.

**Table 1:** Association between sociodemographic features and sentiment in a psychiatric inpatient cohort. Coefficients refer to standard deviation units of positive or negative sentiment.

	Positive Sentiment Score			Negative Sentiment Score		
Feature	coeff.	95% CI	P Value	coeff.	95% CI	P Value
**Age (decade)**	-0.02	[-0.05, 0.01]	0.25	-0.02	[-0.06, 0.009]	0.16
**Male gender**	-0.005	[-0.09, 0.08]	0.91	0.03	[-0.06, 0.12]	0.46
**Race/ethnicity (vs. White)**						
**Black**	0.003	[-0.15, 0.15]	0.97	0.16	[0.003, 0.31]	0.05
**Asian**	-0.002	[-0.22, 0.22]	0.98	0.17	[-0.06, 0.40]	0.14
**Hispanic**	0.29	[0.15, 0.43]	<0.001	0.39	[0.25, 0.54]	<0.001
**Other**	-0.23	[-0.46, -0.01]	0.04	0.02	[-0.21, 0.26]	0.85
**Public insurance**	-0.18	[-0.27, -0.10]	<0.001	-0.08	[-0.17, 0.005]	0.06
**Psychotic disorder**	-0.14	[-0.25, -0.03]	0.01	0.24	[0.12, 0.35]	<0.001
**Log Charlson index**	-0.11	[-0.16, -0.05]	<0.001	-0.12	[-0.18, -0.07]	<0.001
**Admitted via ER**	0.05	[-0.04, 0.14]	0.31	-0.03	[-0.13, 0.06]	0.51

We then examined the predictive validity of sentiment scores for hospital readmission (Table 2). In Cox regression models incorporating all of the features from Table 1, greater positive sentiment was associated with significant reduction in readmission hazard (HR 0.88, 95% CI 0.81–0.96). No significant effect of negative sentiment was identified.

**Table 2:** Cox regression examining association between sentiment and readmission hazard in a psychiatric inpatient cohort.

Feature	HR	95% CI	*P* Value
**Positive sentiment**	0.88	[0.81, 0.96]	0.004
**Negative sentiment**	0.98	[0.90, 1.07]	0.65
**Age (decade)**	0.96	[0.91, 1.02]	0.16
**Male sex**	1.01	[0.87, 1.18]	0.91
**Race/ethnicity (versus White)**			
**Black**	1.17	[0.89, 1.53]	0.26
**Asian**	0.86	[0.59, 1.25]	0.42
**Hispanic**	0.84	[0.66, 1.08]	0.18
**Other**	0.42	[0.24, 0.74]	0.002
**Insurance (public)**	0.58	[0.50, 0.67]	<0.001
**Psychotic disorder**	1.01	[0.83, 1.23]	0.91
**Log Charlson Index**	1.58	[1.43, 1.75]	<0.001
**Admitted via ER**	0.98	[0.84, 1.14]	0.78

### Medical Cohort

The second cohort was drawn from discharges from a general internal medicine service during the same time period. In total, this cohort included 15,011 individuals with 20,859 discharges (S1 Table)—mean 1.39 discharges (SD 1.08) per patient, 79.0% with a single discharge during the study period. Mean age was 62.3 (SD 18.9); mean Charlson comorbidity index was 8.0 (SD 5.8). The cohort was 54.0% male and 78.5% Caucasian, 7.2% black, 3.2% Asian, 7.1% Hispanic, and the remainder other or unreported race. Public insurance was listed in 61.2% of admissions, and 45.0% were admitted from the emergency department rather than directly to the medical unit.

Association between individual clinical features and positive or negative sentiment, adjusted for all other features, is listed in Table 3. As with psychiatric admissions, comorbidity was associated with decreased negative and positive sentiment; greater age was also associated with decrease in both types of sentiment. Among medical admissions, unlike psychiatric admissions, no effects of race or insurance type were identified. Once again, presence of greater levels of positive sentiment was associated with reduced readmission hazard in a Cox regression model (HR 0.88, 95% CI 0.85–0.92, Table 4), with no significant effect observed for negative sentiment. Similarly, greater levels of positive but not negative sentiment were associated with reduced mortality risk in Cox regression model (HR 0.80, 95% CI 0.74–0.86, Table 5). Differences by principal admitting diagnosis (grouped by category) are illustrated in S1 Fig and S2 Table.

**Table 3:** Association between sociodemographic features and sentiment in a general medical cohort.

Feature	Positive sentiment score			Negative sentiment score		
	coeff.	95% CI	P Value	coeff.	95% CI	P Value
**Age (decade)**	-0.02	[-0.03, -0.006]	0.002	-0.03	[-0.04, -0.02]	<0.001
**Male gender**	-0.003	[-0.03, 0.03]	0.83	0.006	[-0.02, 0.04]	0.69
**Race/ethnicity (vs. White)**						
**Black**	-0.03	[-0.09, 0.03]	0.29	-0.01	[-0.07, 0.05]	0.75
**Asian**	0.02	[-0.07, 0.11]	0.69	0.02	[-0.07, 0.11]	0.67
**Hispanic**	-0.006	[-0.07, 0.05]	0.85	0.03	[-0.03, 0.09]	0.33
**Other**	-0.02	[-0.10, 0.06]	0.67	0.07	[-0.01, 0.15]	0.10
**Public insurance**	-0.003	[-0.03, 0.03]	0.85	-0.003	[-0.03, 0.03]	0.86
**Log Charlson index**	-0.07	[-0.09, -0.05]	<0.001	-0.07	[-0.09, -0.04]	<0.001
**Admitted via ER**	0.03	[0.00, 0.06]	0.05	0.01	[-0.02, 0.04]	0.40

**Table 4:** Cox regression examining association between sentiment and readmission hazard in a general medical cohort.

Feature	HR	95% CI	P Value
**Positive sentiment**	0.88	[0.85, 0.92]	<0.001
**Negative sentiment**	0.98	[0.95, 1.01]	0.12
**Age (decade)**	0.97	[0.95, 0.99]	<0.001
**Male sex**	1.06	[1.01, 1.11]	0.01
**Race/ethnicity (versus White)**			
**Black**	0.96	[0.88, 1.06]	0.45
**Asian**	0.71	[0.61, 0.82]	<0.001
**Hispanic**	0.99	[0.91, 1.08]	0.85
**Other**	0.82	[0.72, 0.93]	0.002
**Insurance (public)**	0.45	[0.43, 0.47]	<0.001
**Log Charlson Index**	1.91	[1.83, 1.99]	<0.001
**Admitted via ER**	1.02	[0.98, 1.07]	0.27

**Table 5:** Cox regression examining association between sentiment and mortality in a general medical cohort.

Feature	HR	95% CI	P Value
**Positive sentiment**	0.80	[0.74, 0.86]	<0.001
**Negative sentiment**	1.02	[0.97, 1.08]	0.36
**Age (decade)**	1.30	[1.25, 1.35]	<0.001
**Male sex**	1.26	[1.14, 1.39]	<0.001
**Race/ethnicity (versus White)**			
**Black**	0.78	[0.62, 0.98]	0.03
**Asian**	0.78	[0.58, 1.06]	0.11
**Hispanic**	0.62	[0.47, 0.81]	0.001
**Other**	0.96	[0.75, 1.24]	0.77
**Insurance (public)**	1.05	[0.97, 1.13]	0.20
**Log Charlson Index**	2.19	[1.97, 2.42]	<0.001
**Admitted via ER**	0.82	[0.76, 0.88]	<0.001

## Discussion

We measured sentiment in more than 17,000 patients with 23,000 hospital discharges across two clinical cohorts. In general, notes for individuals with a greater burden of medical illness included less sentiment, both positive and negative. Interestingly, among psychiatric patients, public insurance was associated with less positive sentiment, while notes for Hispanic patients included greater levels of both positive and negative sentiment. In both cohorts, positive sentiment predicted reduced readmission hazard, with ~12% decrease per standard deviation above the mean sentiment value in the cohort; in the general medical cohort, we also observed association with reduced mortality—for one standard deviation increase in positive sentiment, mortality hazard was reduced by ~20%. (While outcome prediction would undoubtedly be improved with more complex models incorporating individual diagnoses and medications, the intention in the present study was simply to demonstrate the informativeness of a novel derived data type, while controlling for standard sociodemographic variables and an aggregate measure of clinical severity, the Charlson comorbidity index.)

These results are particularly notable given that the sentiment-scoring system was not derived with any particular eye towards clinical documentation or medical vocabulary. As such, a key question is what such sentiment scores actually reflect; we employ the term 'sentiment' throughout simply for consistency with the descriptions of this approach in the machine learning literature.

In part, these measures may aggregate symptoms or diagnoses reflecting negative valence—depression, anxiety, and pain, for example. In this context, they represent simply an efficient general-purpose algorithm for aggregating clinical features not fully explained by ICD9 codes alone, consistent with dimensional models of psychopathology[23]. On the other hand, the positive sentiment domain is less likely to be explainable solely in terms of symptoms or disorders.

An additional possibility is that some of the signal we detect actually reflects attitudes toward particular patients. For example, individuals with more unstable or difficult-to-control illness might induce stronger emotions among clinicians, contributing to more use of affectively-loaded terms. Whether this reflects a simple marker of disease severity or actually influences treatment outcomes merits further study. Our results should be taken as an indication that this line of inquiry is likely to be productive, and as proof of principle rather than a definitive investigation of a single phenomenon.

A key limitation of our data is the inability to characterize the physicians who write the individual notes. Thus, while we can account for patient-level differences, we were not able to examine clinician-level differences. With sufficient data regarding individual clinicians, sentiment measures would provide an opportunity to further investigate clinician-level factors that may influence outcomes.

Several other caveats must be considered. First, these results represent only one possible approach to identifying sentiment, which is an area of active investigation within computer science. Therefore, it is possible—indeed, likely—that other approaches could yield different results, depending on the corpus used to develop sentiment measures and the means of classification employed[2]. Second, further studies addressing portability of this approach will be needed, although our observation of similar effects in two distinct clinical cohorts provides a reason for optimism. Third, our readmission data are subject to bias because this health care system is not 'closed'—individuals may be readmitted to other hospitals, for example. On the other hand, this omission would most likely bias our results towards the null. Moreover, the detection of similar effects on overall mortality, for which complete data are available, provides further support for the predictive validity of sentiment. Examination of these tools in those few closed systems for which both narrative notes and claims codes are available will be helpful in this regard.

Among our specific findings, the observation that public insurance is associated with diminished positive sentiment in psychiatric discharges, but not general medical discharges, merits further consideration. Insurance type strongly correlates with socioeconomic status and with burden of stressors; it is possible that documentation of these stressors is detected as less positive sentiment, although we would expect to see greater negative sentiment. Alternatively, in Massachusetts, so-called managed behavioral health has led to substantial challenges in finding care for psychiatric patients, which may also be reflected in clinician sentiment. In general, physicians caring for medical patients in this hospital system have little awareness of insurance type, while those caring for psychiatric patients are reminded on a daily basis of insurance.

Yet another intriguing observation among psychiatric patients, but not general medical patients, is that Hispanic ethnicity is associated with increased sentiment, both positive and negative. While this finding could arise from greater need for an interpreter (for example, if the interpreter introduces more subjective language), we would then expect to see differences for other non-White groups, as this medical center treats a large number of non-English-speaking Asian patients as well. Alternatively, a growing literature suggests important cultural differences in symptom expression. In one recent study, the nature and understanding of psychotic-like symptoms in Latino patients differed from comparison groups[24]. Differences in symptoms appear to be substantial enough to impact validity of diagnostic criteria in psychiatry, as an investigation in adolescents suggested[25].

A number of possible next steps could be considered based upon these results. First, if portability of these results is established in other health systems, sentiment might contribute to clinically-useful automated prediction models to target strategies aimed at preventing readmission, and compared directly to clinician predictions at time of discharge. Second, more generally, understanding the sentiment expressed in narrative clinical notes may help to elucidate patient- or clinician-level factors associated with differential outcomes. These factors could then be targeted in an effort to modify those outcomes. At minimum, the present results demonstrate the feasibility and potential utility of a novel high-throughput approach to characterizing narrative notes capable of spanning broad clinical populations.

## Supporting Information

S1 Fig Mean positive (upper panel) and negative (lower panel) sentiment, by primary admission diagnosis. (DOCX)

S1 Table Descriptive characteristics of two outcome cohorts. (DOCX)

S2 Table Primary admission diagnoses with greatest negative and positive sentiment scores. (DOCX)
