# Supplementary material for: Sentiment Measured in Hospital Discharge Notes Is Associated with Readmission and Mortality Risk: An Electronic Health Record Study
Source: PLoS One. 2015 Aug 24;10(8):e0136341. doi: 10.1371/journal.pone.0136341 (PMC4547711; doi:10.1371/journal.pone.0136341)

**S1 Fig. Mean positive (upper panel) and negative (lower panel) sentiment, by primary admission diagnosis**


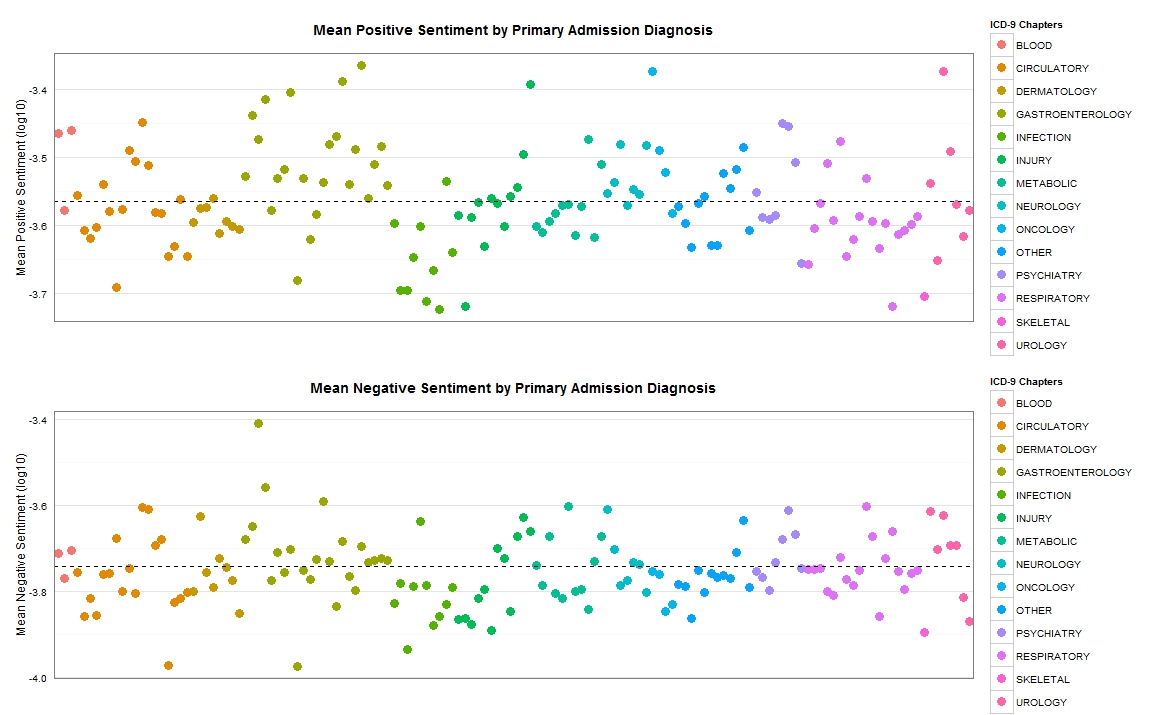

Supplement: S1 Fig — (DOCX) [file pone.0136341.s001.docx]
