# Supplementary material for: Sentiment Measured in Hospital Discharge Notes Is Associated with Readmission and Mortality Risk: An Electronic Health Record Study
Source: PLoS One. 2015 Aug 24;10(8):e0136341. doi: 10.1371/journal.pone.0136341 (PMC4547711; doi:10.1371/journal.pone.0136341)
Supplement: S1 Table — (DOCX) [file pone.0136341.s002.docx]

**S1 Table. Descriptive characteristics of two outcome cohorts**

|  | **Inpatient psychiatric cohort (n=2010)** | | **Internal medicine cohort (n=15011)** | |
| --- | --- | --- | --- | --- |
| **Feature** | **N** | **Pct** | **N** | **Pct** |
| Gender (male) | 984 | 49.1% | 8107 | 54.0% |
| Race (white) | 1433 | 71.3% | 11780 | 78.5% |
| Insurance (public) | 1097 | 54.6% | 9187 | 61.2% |
| Psychotic disorder | 379 | 18.9% | - | - |
|  |  | |  | |
