# Supplementary material for: Sentiment Measured in Hospital Discharge Notes Is Associated with Readmission and Mortality Risk: An Electronic Health Record Study
Source: PLoS One. 2015 Aug 24;10(8):e0136341. doi: 10.1371/journal.pone.0136341 (PMC4547711; doi:10.1371/journal.pone.0136341)
Supplement: S2 Table — (DOCX) [file pone.0136341.s003.docx]

**S2 Table. Primary admission diagnoses with greatest negative and positive sentiment scores**

**a. Negative sentiment**

| **Rank** | **ICD-9 Chapter** | **PheWAS Code** | **PheWAS Description** | **Negative Sentiment Score (log10)** |
| --- | --- | --- | --- | --- |
| 1 | GASTROENTEROLOGY | 555.2 | Ulcerative colitis | -3.41 |
| 2 | GASTROENTEROLOGY | 558 | Noninfectious gastroenteritis | -3.56 |
| 3 | GASTROENTEROLOGY | 573.4 | Acute and subacute necrosis of liver | -3.59 |
| 4 | RESPIRATORY | 499 | Cystic fibrosis | -3.60 |
| 5 | METABOLIC | 264.2 | Failure to thrive | -3.60 |
| 6 | CIRCULATORY | 427.7 | Tachycardia NOS | -3.61 |
| 7 | NEUROLOGY | 320 | Meningitis | -3.61 |
| 8 | CIRCULATORY | 427.9 | Palpitations | -3.61 |
| 9 | PSYCHIATRY | 317 | Alcohol-related disorders | -3.61 |
| 10 | UROLOGY | 585.1 | Acute renal failure | -3.62 |
|  |  |  |  |  |
| **b. Positive Sentiment** | | | | |
| **Rank** | **ICD-9 Chapter** | **PheWAS Code** | **PheWAS Description** | **Positive Sentiment Score (log10)** |
| 1 | GASTROENTEROLOGY | 577.2 | Chronic pancreatitis | -3.37 |
| 2 | ONCOLOGY | 155.1 | Hepatic cancer, primary | -3.37 |
| 3 | UROLOGY | 585.31 | Renal dialysis | -3.37 |
| 4 | GASTROENTEROLOGY | 575.1 | Cholangitis | -3.39 |
| 5 | INJURY | 979 | Adverse drug events and drug allergies | -3.39 |
| 6 | GASTROENTEROLOGY | 562.2 | Diverticulitis | -3.41 |
| 7 | GASTROENTEROLOGY | 558 | Noninfectious gastroenteritis | -3.42 |
| 8 | GASTROENTEROLOGY | 555.1 | Crohn's disease | -3.44 |
| 9 | CIRCULATORY | 427.7 | Tachycardia NOS | -3.45 |
| 10 | PSYCHIATRY | 316 | Substance addiction and disorders | -3.45 |
